# The effect of FTO gene rs9939609 polymorphism on the association between colorectal cancer and different types of dietary fat intake: a case-control study

**DOI:** 10.1186/s40101-023-00333-4

**Published:** 2023-08-05

**Authors:** Azadeh Hajipour, Naeemeh Hassanpour Ardekanizadeh, Zahra Roumi, Soheila Shekari, Bahareh Aminnezhad Kavkani, Seyedeh Hayedeh Mousavi Shalmani, Bojlul Bahar, Shirin Tajadod, Marjan Ajami, Ghasem Azizi Tabesh, Maryam Gholamalizadeh, Saeid Doaei

**Affiliations:** 1 School of Health, Qazvin University of Medical Sciences, Qazvin, Iran; 2 Torbat Jam Faculty of Medical Sciences, Torbat Jam, Iran; 3 Department of Nutrition, Science and Research Branch, Islamic Azad University, Tehran, Iran; 4 Guilan University of Medical Sciences, Rasht, Iran; 5 Nutrition Sciences and Applied Food Safety Studies, Research Centre for Global Development, School of Sport & Health Sciences, University of Central Lancashire, Preston, UK; 6 Department of Nutrition, School of Public Health, International Campus, Iran University of Medical Sciences, Tehran, Iran; 7 Department of Food and Nutrition Policy and Planning National Nutrition and Food Technology Research Institute School of Nutrition Sciences and Food Technology, Shahid Beheshti University of Medical Sciences, Tehran, Iran; 8 Genomic Research Center, Shahid Beheshti University of Medical Sciences, Tehran, Iran; 9 Cancer Research Center, Shahid Beheshti University of Medical Sciences, Tehran, Iran; 10 Department of Community Nutrition, Faculty of Nutrition and Food Technology, National Nutrition and Food Technology Research Institute, Shahid Beheshti University of Medical Sciences, Tehran, Iran

**Keywords:** Colorectal cancer, Fat mass and obesity-associated (FTO) gene, Dietary fats, Polymorphism, Genotype, Oleic acid

## Abstract

**Background:**

Colorectal cancer (CRC) is one of the most common cancers in the world. Some dietary factors such as fat intake have been identified as the risk factors for CRC. This study aimed to investigate the effect of fat mass and obesity-associated (FTO) gene rs9939609 polymorphism on the association between CRC and different types of dietary fats.

**Methods:**

This case-control study was performed on 135 CRC cases and 294 healthy controls in Tehran, Iran. Data on demographic factors, anthropometric measurements, physical activity, the intake of different types of dietary fats, and FTO gene rs9939609 polymorphism was collected from all participants. The association between cancer and dietary fat intake in individuals with different FTO genotypes was assessed using different models of logistic regression.

**Results:**

Oleic acid intake was higher in the case group compared to the control group in both people with TT (7.2±3.46 vs. 5.83±3.06 g/d, *P*=0.02) and AA/AT genotypes (8.7±6.23 vs. 5.57 ±3.2 g/d, *P*<0.001). Among carriers of AA/AT genotypes of FTO rs9939609 polymorphism, a positive association was found between CRC and higher intakes of oleic acid (OR=1.12, CI95% 1.03–1.21, *P*=0.01) and cholesterol (OR=1.01, CI95% 1.00–1.02; *P*=0.01) after adjusting for age, sex, physical activity, alcohol use, smoking, calorie intake, and body mass index.

**Conclusion:**

Higher intakes of cholesterol and oleic acid were associated with a higher risk of CRC in FTO-risk allele carriers. The association of CRC and dietary fat may be influenced by the FTO genotype. Further longitudinal studies are warranted to confirm these findings.

## Introduction

Colorectal cancer (CRC) is the third most common cancer worldwide [1]. The incidence of CRC is continuously increasing in developing countries [2, 3]. In Iran, CRC is the third most prevalent cancer in women and the fourth most prevalent in men [4].

Genetics and environmental factors have been identified as the most important risk factors for CRC [5]. Among several environmental factors, a diet rich in fat may have a key role in the increased incidence of CRC [6]. In some studies, ω-6 polyunsaturated fatty acids (PUFAs) such as linoleic acid (LA, 18:2ω-6) and arachidonic acid (ARA, 20:4 ω-6) have been reported as an enhancer of tumorigenesis, inflammation, and CRC development [7]. Also, a recent study reported dietary trans fatty acids (TFA), particularly elaidic acid, are positively related to rectal cancer mainly because they may lead to a higher inflammation and oxidative stress by irritation of the colon and rectal mucosa [8]. Some other studies indicated a positive relationship between dietary intake of saturated fatty acids (SFA) and increased risk of CRC [9]. However, some types of ω-3 polyunsaturated fatty acids, such as C20:5 ω-3 eicosapentaenoic acid (EPA) and C22:6 ω-3 docosahexaenoic acid (DHA), may have anti-CRC effects [10, 11]. Furthermore, oleic acid may have some beneficial effects against CRC [12].

On the other hand, obesity not only increases the risk of chronic diseases such as cardiovascular disease, stroke, and nonalcoholic fatty liver but also increases the risk of cancer [13]. Recent studies examined the interactions between obesity, CRC, and obesity-related genetic factors such as fat mass and obesity-associated protein (FTO) genes [3, 14]. The FTO gene is noticeably expressed in the hypothalamus and is associated with the regulation of food intake and energy balance [15]. FTO inhibitors such as Meclofenamic acid (MA), FB23-2, and R2 hydroxyglutarate (R2HG) have been recently investigated as anticancer drugs in many types of cancer [16]. The mechanism of the effect of the FTO gene on CRC is not well understood. A previous study has reported the role of adenosine monophosphate-activated protein kinasealpha2 (AMPKα2) in suppressing FTO and inducing apoptosis [17]. Another recent study indicated that FTO and cmyc protooncogene (MYC) genes might also be responsible for enhancing the proliferative and migratory functions of CRC cells [18].

Due to the high prevalence of CRC and the lack of adequate research on the possible association of CRC with the FTO gene and dietary fat intake, this case-control study aimed to assess the effect of FTO gene rs9939609 polymorphism on the association between CRC and different types of dietary fat intake in the Iranian adults.

## Methods

This study was a case-control carried out on 135 CRC cases and 294 healthy controls referred to Firoozgar Hospital, Tehran, Iran. The inclusion criteria for the case group included age between 35 and 70 years, not more than 6 months after the CRC diagnosis, and willingness to participate in this study. The inclusion criteria for the control group included people with age between 35 and 70 years, no history of malignancy, lack of diseases affecting dietary intake, do not take drugs that affect body weight, and willingness to participate in this study. Innitially, 540 people (180 patients with CRC and 360 healthy people matched in terms of age and sex) were included. Some participants were excluded due to lack of access to required data (*n*=62), consumption of fatty acid supplements and nutritional supplements containing fat in the last year (*n*=38), and diagnosis of malignancy during the study in the control group (*n*=11). All participants were explained the details of the present study, and written consent was obtained prior to the data collection.

Socio-demographic information of participants including age, gender, level of education, income level, medical history, and marital and employment status was collected through a face-to-face interview. Height and weight were measured by trained people using SECA stadiometer and Seca scales with an accuracy of 0.5 cm and 100 g, respectively. The patients’ BMI was then calculated by the participants’ weight in kilograms divided by the square of height in meters. A validated International Questionnaire (IPAQ) was used to measure physical activity.

### Dietary assessments

The required dietary information was collected through face-to-face interviews by trained dietitians using a validated semi-quantitative Food Frequency Questionnaire (FFQ) consisting of 168 food items with standard portion sizes commonly consumed by Iranian people [19]. In order to validate of FFQ, twelve repeated 24 h dietary recalls, two FFQ, biochemical markers in serum, and urine samples were used to assess the validity and the results indicated that the FFQ has reasonable relative validity and reliability for nutrient intakes in Tehranian adults and appears to be an acceptable tool for assessing nutrient intakes in this population [20]. The amount of intake of different dietary fats, including saturated fatty acids, trans and unsaturated fatty acids, ω-3 fatty acids, and ω-6 fatty acids was estimated using Nutritionist IV software. Daily intakes of dietary fats were calculated for each participant by using the US Department of Agriculture food consumption database, which was modified for Iranian foods. The USDA provide the basic foundation for food and nutrition investigations and monitoring, policy, and dietary practice.

### Genotyping

Five milliliters of the participants’ blood samples was collected at the beginning of the study into ethylenediaminetetraacetic acid (EDTA) tubes. The samples were transferred to the cellular and molecular laboratory of Shahid Beheshti University of Medical Sciences, Tehran, Iran. The PCR products were examined to identify rs9939609 polymorphism of the FTO gene by the Tetraprimer amplification refractory mutation system (ARMS) PCR method.

Blood samples were washed with (Tris-HCl 10 mmol/L, MgCl2 5 mmol/L, lysis buffer Triton X% 1 pH 7.6), and phosphate buffer saline (PBS) and red blood cell (RBCs) were removed from the method. The DNA extraction kit manufactured by GeneAll (GeneAll Biotechnology Co. Ltd, Korea) was used to extract and purify DNA samples. After determining the DNA concentration, it was kept at a suitable temperature for the next steps. To design the primers required to perform the PCR reaction, the sequence of adjacent single nucleotide polymorphism (SNP) target regions was extracted, and the appropriate primers were designed by referring to the dbSNP database located at http://www.ncbi.nlm.nih.gov/SNP (Table 1). Primers designed by the Basic Local Alignment Search Tool (BLAST) Basic Local Alignment Search Tool service on the National Center for Biotechnology Information (NCBI) site were also evaluated to find homologous regions in the human genome. The desired SNPs were genotyped by allele-specific primers according to the last nucleotide at the end of '3 primers and complementation of the SNP site, using Tetraprimer the Amplification Refractory Mutation System polymerase chain reaction (Tetra -ARMS PCR). This technique required four primers to genetically amplify a large fragment of the template DNA containing the SNP (control fragment) and two smaller fragments representing each of the two allele products. The primers were designed to produce allelic amplified fragments of different sizes and distinguished by agarose gel electrophoresis. To increase the specificity of the reaction, in addition to the first nonpairing at the end of the '3 specific internal primers, an additional pairing was intentionally created at the third position of the '3' end of each of the two internal specific primers. For specific amplification of fragments around the desired polymorphism, 25 μl PCR including 200 ng DNA, 3 μl 10X PCR buffer, 1.5 μl d10TP 10 mM, 1 μl 250 MgCl250 mM, 1.5 pH Internal go (OR and IF), 1 picomole of internal return primer (IR), 0.5 picomoles of external go primer (OF), and 0.4 μl (2 units) of Taq DNA polymerase (synagen) by PCR (model thermocycler) Eppendorf, Germany). The optimum temperature schedule for PCR was as follows: initial melting step 3 min, 30 temperature repetitions with denaturation for 30 s, 45 s for binding the primers, propagation temperature for 55 s, and eventually, and 10 min at room temperature were selected for final propagation. PCR products were then electrophoresed on٪ 1 agarose gel for 40 min at 80 volts, and finally, the separated bands were observed and analyzed by UV Gel Documentation. The sequences for the primers are presented in (Table 1).

**Table 1:** The sequences of the primers used for tetraprimer amplification refractory mutation system polymerase chain reaction (T-ARMS-PCR)

Gene	Primer sequence	Product size	Annealing temp. °C
FTO	Forward outer primer: AGTTCCAGTCATTTTTGACAGC Reverse outer primer: AGCCTCTCTACCATCTTATGTC Forward inner primer: CCTTGCGACTGCTGTGAATATA Reverse inner primer: GAGACTATCCAAGTGCATCTCA	Control fragment: 429bp T allele: 278bp A allele: 194bp	59.50

### Statistical analysis

The normal distribution of data was confirmed using Kolmogorov–Smirnov normal distribution Test. General characteristics and dietary intake of the case and control groups were compared by chi-square (for qualitative variables) and independent *t* test (for quantitative variables) methods. Independent tests were used because people with different FTO genotypes (which were unmatched) were compared in the matched case and control groups. A simple dominant model was used because it can cover a wide range of plausible genotype-phenotype relationships [21, 22]. The association between CRC and the intake of dietary fats in individuals with different FTO gene genotypes was assessed using different models of logistic regression after adjusting the covariates as follows: Model 1: crude; Model 2: adjusted for age and sex (the male group was the reference group); Model 3: adjusted for age, sex, physical activity, alcohol use (no drink group was the reference group), and smoking (no smoking group was the reference group); and Model 4: adjusted for age, sex, physical activity, alcohol use, smoking, calorie intake, and BMI. All statistical analyses were performed using SPSS software version 23, and *P*˂0.05 was considered statistically significant.

## Results

The general characteristics of the patients and healthy participants included in this study are presented in Table 2. In TT FTO rs9939609 genotype carriers, the mean BMI of the case group was significantly higher than the control group 29±3.9 vs. 27±3.31 kg/m^2^, *P*= 0.02). In the AA/AT dominant genetic model, the cases had higher age, smoking rate, weight, and BMI compared with the control group (All *P*˂0.05). However, the controls had higher heights than the cases. According to the results in the TT genotype, the only significant factor is the BMI, in which the cases had a higher amount of BMI compared to the controls (*P*<0.02). The two groups also did not differ significantly in terms of gender, alcohol consumption, and physical activity in both types of genetic models.

**Table 2:** General characteristics of the case and control groups based on FTO rs9939609 genotype

	TT genotype (*n*=154)			AA/AT genotype (*n*=275)		
	Cases (*n*= 46)	Controls (*n*= 108)	*P*	Cases (*n*= 89)	Controls (*n*= 186)	*P*
Age (years)	51.22±16.08	47.37±10.70	0.141	52.50±17.19	48.07±11.19	<0.012
Gender						
Males (*n*)	25 (54.7%)	41 (47.6%)	0.082	58 (64.6%)	106 (57.3%)	0.201
Females (*n*)	21 (45.3%)	57 (52.4%)		31 (35.4%)	80 (42.7%)	
Alcohol use (*n*)	15 (33%)	5 (5%)	0.061	9 (10.5%)	14 (7.6%)	0.462
Smoking (*n*)	3 (6%)	9 (8.6%)	0.594	37 (42%)	24 (13%)	<0.013
Weight (kg)	70.85±10.26	68.82±7.86	0.183	70.03±10.89	69.62±9.08	0.031
Height (cm)	155.83± 4.72	157.83 ± 6.87	0.082	156.2 ± 5.71	158.65 ± 7.81	0.022
BMI (kg/m^2^)	29.18 ± 3.90	27.67 ± 3.31	0.022	28.68 ± 4.06	27.58 ± 3.25	0.043
Physical activity (hours/week)	7.37 ± 1.84	7.30 ± 1.64	0.831	7.61 ± 2.02	7.33 ± 1.54	0.114

*BMI* Body mass index

The results of measuring the fat consumption in the case and control groups based on the FTO rs9939609 genetic model are presented in Table 3. Significant differences were found in polyunsaturated fat and oleic acid intake in the TT genotype. Polyunsaturated fat intake was higher in the control group, while oleic acid intake was higher in the case group. In addition, the AA/AT genotype carriers of the case group had more consumption of oleic acid and DHA than the controls.

**Table 3:** Fat intake of the case and control groups based on FTO rs9939609 genotype

	TT genotype			AA/AT genotype		
	Cases	Controls	*P*	Cases	Controls	*P*
Cholesterol (mg/d)	267.99 ± 46.32	265.09 ± 47.07	0.721	274.79 ± 56.80	251.8 ± 65.90	0.743
SFA (g/d)	31.20 ± 4.87	32.03 ± 6.67	0.391	31.75 ± 6.39	31.03 ± 11.85	0.582
PUFas (g/d)	15.38 ± 3.72	16.88 ± 4.93	0.402	15.49 ± 4.80	16.39 ± 3.75	0.512
Oleic acid (g/d)	7.20 ± 3.46	5.83 ± 3.06	0.023	8.70 ± 6.23	5.57 ± 3.20	<0.011
Linoleic (g/d)	6.69 ± 2.63	6.33 ± 3.85	0.504	7.19 ± 4.68	5.82 ± 3.19	0.242
α-Linolenic acid(g/d)	0.88 ± 0.84	0.95 ± 0.77	0.614	0.82 ± 0.78	1.01 ± 0.72	0.124
DHA (g/d)	0.07 ± 0.06	0.06 ± 0.05	0.645	0.07 ± 0.06	0.06 ± 0.05	0.313

*SFA* Saturated fatty acids, *DHA* Docosahexaenoic acid, *PUFAs* Polyunsaturated fatty acids

The association between CRC and dietary intake among people with TT FTO rs9939609 genotype was investigated by logistic regression, and the results are presented in Table 4. No association was found between the risk of CRC with the intake of different types of dietary fats (Model 1). The results did not change after adjustment for age and sex (Model 2), further adjustment for physical activity, alcohol use, and smoking (Model 3), and additional adjustment for calorie intake and BMI (Model 4).

**Table 4:** Logistic regression of the association between colorectal cancer and dietary intake among people with TT FTO rs9939609 genotype

	Model 1		Model 2		Model 3		Model 4	
	OR (CI95%)	*P*	OR (CI95%)	*P*	OR (CI95%)	*P*	OR (CI95%)	*P*
Total fat	0.94 (0.89–1.01)	0.051	0.94 (0.88–1.00)	0.071	0.94 (0.88–1.00)	0.07	0.94 (0.88–1.01)	0.122
Cholesterol	0.94 (0.99–1.01)	0.552	0.99 (0.098–1.01)	0.872	0.99 (0.098–1.01)	0.87	0.99 (0.98–1.01)	0.651
SFA	1.01 (0.92–1.09)	0.862	1.01 (0.91–1.11)	0.873	1.01 (0.91–1.11)	0.87	1.01 (0.92–1.12)	0.751
MUFAs	1.00 (0.88–1.09)	0.771	0.96 (0.84–1.09)	0.584	0.96 (0.84–1.09)	0.57	0.97 (0.85–1.31)	0.652
PUFAs	0.98 (0.87–1.18)	0.831	0.99 (0.83–1.17)	0.911	0.99 (0.83–1.17)	0.91	0.98 (0.82–1.17)	0.843
Oleic acid	1.01 (0.98–1.27)	0.811	1.09 (0.94–1.27)	0.242	1.09 (0.94–1.27)	0.24	1.06 (0.90–1.25)	0.431
Linoleic	1.10 (0.96–1.28)	0.163	1.07 (0.90–1.27)	0.401	1.07 (0.90–1.27)	0.40	1.10 (0.92–1.31)	0.262
Linolenic acid	0.99 (0.60–1.63)	0.982	1.10 (0.61–1.01	0.744	1.10 (0.61–1.01	0.74	1.07 (0.58–1.99)	0.80
EPA	1.28 (0.01–2.76)	0.512	8.87 (0.01–3.16)	0.313	8.87 (0.01–3.16)	0.01	4.59 (0.01–24.71)	0.353
DHA	2.88 (.04–18.2)	0.313	1.43 (0.01–29.27)	0.934	1.43 (0.01–29.27)	0.93	3.67 (0.01–10.10)	0.751

Model 1: crude. Model 2: adjusted for age and sex. Model 3: adjusted for age, sex, physical activity, alcohol use, and smoking. Model 4: adjusted for age, sex, physical activity, alcohol use, smoking, calorie intake, and BMI

*SFA* Saturated fatty acids, *EPA*, Eicosapentaenoic acid, *DHA*, Docosahexaenoic acid, *MUFAs*, monounsaturated fatty acids, *PUFAs*, Polyunsaturated fatty acids

The results of logistic regression on the interactions between CRC and dietary intake among people with AA/AT FTO rs9939609 genotype are reported in Table 5. The risk of CRC was significantly associated with the dietary intake of cholesterol and oleic acid (Model 1). The results did not change after adjustment for age and sex (Model 2), further adjustment for physical activity, alcohol use, and smoking (Model 3). However, no significant association between CRC risk and oleic acid was found after further adjustment for calorie intake and BMI.

**Table 5:** Logistic regression of the association between colorectal cancer and dietary intake among people with AA/AT FTO rs9939609 genotype

	Model 1		Model 2		Model 3		Model 4	
	OR (CI95%)	*P*	OR (CI95%)	*P*	OR (CI95%)	*P*	OR (CI95%)	*P*
Total fat	0.99 (0.95–1.02)	0.552	0.98 (0.95–1.02)	0.551	0.98 (0.95–1.02)	0.552	1.01 (0.96–1.06)	0.522
Cholesterol	1.01 (1.00–1.02)	0.014	1.01 (1.00–1.02)	0.041	1.01 (1.00–1.02	0.043	1.01 (1.00–1.02)	0.037
SFA	0.95 (0.90–1.01)	0.131	0.96 (0.91–1.03)	0.304	0.96 (0.91–1.03)	0.303	0.99 (0.92–1.07)	0.938
MUFAs	1.00 (0.93–1.07)	0.957	1.01 (0.93–1.09)	0.752	1.01 (0.93–1.09)	0.751	1.04 (0.96–1.14)	0.282
PUFAs	0.90 (0.82–1.00)	0.051	0.90 (0.82–1.00)	0.052	0.90 (0.82–1.00)	0.054	0.92 (0.83–1.02)	0.112
Oleic acid	1.12 (1.03–1.21)	0.008	1.11 (1.02–1.25)	0.012	1.11 (1.02–1.25)	0.011	1.08 (0.98–1.19)	0.091
Linoleic acid	1.11 (0.98–1.21)	0.049	1.13 (0.98–1.25)	0.048	1.13 2(0.98–1.25)	0.06	1.11 (0.99–1.23)	0.052
α-Linolenic acid	1.11 (0.74–1.65)	0.612	1.12 (0.71–1.77)	0.601	1.12 (0.71–1.77)	0.603	1.22 (0.76–1.94)	0.411
EPA	1.01 (0.01–5.43)	0.087	1.01 (0.01–5.54)	0.091	1.01 (0.01–5.63)	0.092	1.01 (0.01–5.78)	0.153
DHA	6.97 (0.6–13.27)	0.424	2.02 (0.12–6.62)	0.221	2.02 (0.12–6.62)	0.218	3.10 (13–4.61)	0.211

Model 1: crude. Model 2: adjusted for age and sex. Model 3: adjusted for age, sex, physical activity, alcohol use, and smoking. Model 4: adjusted for age, sex, physical activity, alcohol use, smoking, calorie intake, and BMI

*SFA* Saturated fatty acids, *EPA*, Eicosapentaenoic acid, *DHA* Docosahexaenoic acid, *MUFAs* Monounsaturated fatty acids, *PUFAs* Polyunsaturated fatty acids

## Discussion

The obtained results from this study showed CRC patients in the AA/AT genetic model had higher age, smoking rate, and weight compared with the control group. Our results were in line with the results of several studies that have shown a weight gain, smoking, and advanced age were positively associated with CRC risk [23–26]. Moreover, in both genetic model groups, BMI was greater in the cases. Previous studies consistently reported that overweight and/or obesity was positively associated with CRC [27, 28]. In the present study, controls of the AA/AT genotype group significantly were taller than the cases. However, studies have shown that greater height positively affects the chance of CRC [29–32]. Explaining this association, researchers suggested reasons such as the higher length of the intestines and the greater number of cells; therefore, taller people might have a higher risk of cell mutations [32]. This contradiction in the findings can be the result of the differences in the number, ethnicity/race, and nutritional status of the study subjects.

According to studies, the FTO gene was related to obesity and certain types of malignancies, including CRC, by altering the impacts of leptin and adiponectin on colorectal carcinogenesis or participating in adipogenesis and tumorigenesis through the mammalian target protein rapamycin (mTOR). FTO inhibitors have also been reported as antiobesity and anticancer factors in vivo [33, 34]. Furthermore, our previous study identified a significant positive association of the A allele with the risk of developing CRC in Iranians [35].

In our study, the cases in both genotype groups significantly consumed higher oleic acid. Also, dietary intake of oleic acid was positively associated with CRC in the AA/AT genotype group. Although dietary fat intake has been related to increasing CRC risk, finding out specific FA associations and their mechanistic basis was challenging [36, 37]. Several studies have shown a relationship between serum levels of specific fatty acids with CRC [38, 39]. In line with our findings, a recent study has reported that oleic acid can promote CRC metastasis. NADPH oxidase 4 (NOX4) expressions and reactive oxygen species (ROS) production can be affected by increased angiopoietin-like 4 (ANGPTL4) secretion induced by oleic acid. Also, oleic acid provokes the expression of the lipoprotein lipase inhibitor ANGPTL4 and prevents lipid overload in cells resulting in the induction of NOX4 expression. These findings proposed that the tumor cell response to the high level of lipids such as oleic acid via triggering off the ANGPTL4/ NOX4/ROS axis could be one of the underlying causes of CRC metastasis [40]. Numerous studies reported that DHA has antineoplastic properties, particularly in CRC, by altering the morphology of cells to apoptotic forms, disrupting cell connections, and reactivating caspase-3 [41–43].

DHA is a potent agent in decreasing cell number and cell proliferation rate with induction of apoptosis in KRAS mutants. KRAS mutations were found in about 40% of human CRCs that are able to activate chemotherapy-resistant CRC stem cells (CCSCs) [44]. Our findings failed to confirm the result of these studies since DHA consumption was related to CRC in the TT genotype and was higher in the AA/AT cases. This may be due to the FTO gene’s effect on this fatty acid’s role, so more studies need to be done on the relationship of this gene with DHA fatty acid.

The results of this study also demonstrated that in the AA/AT genotype, intake of cholesterol and linoleic was positively associated with CRC. Okuno et al. suggested that linoleic acid enhanced colon carcinogenesis [45]. Also, the results of a metaanalysis showed that there was a positive association between linoleic intake and CRC risk [46]. The linoleic acid metabolic pathway is one of the most influential metabolic pathways in CRC patients and plays an important role in the development of inflammation [47]. Studies have demonstrated that linoleic acid metabolism might be related to Enterobacteriaceae. These bacteria have a role in promoting cancer and are significantly abundant in CRC patients [48]. 12,13-DHOME (isoleucotoxin diol) is one of the metabolites of cytochrome P450 monooxygenase oxidation of linoleic acid, which inhibits the immune system and cell proliferation [49].

Inconsistent with the results, a positive association between cholesterol and CRC risk has previously been reported [50, 51]. The mechanism of the effect of cholesterol on cancer is stated in several studies. Squalene epoxidase (SQLE) was a rate-limiting enzyme in cholesterol biosynthesis. Cholesterol can downregulate SQLE gene expression. Thus, critical cellular checkpoints are eliminated, increasing aberrant cell proliferation [52]. Furthermore, dietary cholesterol induces inflammatory responses by increasing IL-1β, a potent cancer-promoting cytokine [53].

Our results showed that the intake of polyunsaturated fat was higher among controls in the TT genotype. Studies have reported the role of PUFAs, a small group of dietary unsaturated fatty acids, in decreasing tumor development and cancer complaints, especially CRC [54, 55]. According to research, anticarcinogenic effects of PUFAs against CRC were related to different mechanisms such as inducing oxidative stress; altering fluidity, compressibility, fusion, and protein function of cancer cell membranes; reducing inflammation by inhibition of cyclooxygenase (COX) activity; and also modulation of DNA methylation [55, 56]. A review of recent data showed that high amounts of fat induce tumor-promoting activities by altering bile acid metabolism and gut microbiota composition [37]. It is similar to our finding that total fat positively affects CRC.

As a strength, this study was the first study (according to the authors’ information) to investigate the association between different dietary fats and CRC in people with different FTO genotypes. Different types of dietary fats and fatty acids were considered in this study. However, this study had some limitations. First, the sample size was relatively small in the case group. Second, the FFQ was used to collect information about food intake, which is a self-report tool and its accuracy may be questioned.

## Conclusion

Results of the present study showed that the CRC patients with the AA/AT allele of rs9939609 polymorphism are consuming higher oleic acid and DHA. Also, in this group, oleic acid, linoleic, and cholesterol had a positive association with CRC. While the TT genotype cases had a higher intake of oleic acid, the controls consume more PUFA. Furthermore, the total fat and DHA from the diet were related to a higher risk of CRC in the TT genotype. Future comprehensive studies are needed to verify these findings and to address the underlying mechanisms of the difference in the relationship between various types of FTO genotypes and CRC.

## Data Availability

Not applicable

## Abbreviations

CRC: Colorectal cancer
FTO: Fat mass and obesity-associated
BMI: Body mass index
DHA: Docosahexaenoic acid
